# The Effect of Age on Semen Quality Among Male Partners of Infertile Couples: An Observational Study in a Tertiary Care Center in Eastern India

**DOI:** 10.7759/cureus.42882

**Published:** 2023-08-03

**Authors:** Md. Zabihullah, Tribhuwan Kumar, Kamlesh Jha, Kumar Siddharth, Abhimanyu Ganguly, Yogesh Kumar, Raihan Mannan

**Affiliations:** 1 Physiology, All India Institute of Medical Sciences (AIIMS), Patna, IND; 2 Physiology, Netaji Subhas Medical College and Hospital (NSMCH), Patna, IND

**Keywords:** paternal age, sperm concentration, sperm, semen analysis, infertility

## Abstract

Introduction: Male infertility contributes to a significant proportion of infertility cases, and advanced paternal age has been suggested to affect semen quality and fertility. However, the relationship between age and semen quality remains inconclusive, with conflicting findings reported in the literature. This study aimed to investigate the effect of age on semen quality among male partners of infertile couples in a tertiary care center in eastern India.

Methods: A cross-sectional observational study was conducted, involving 390 male participants aged 21-50 years, who were referred to the andrology laboratory for semen analysis between January 2019 and December 2022. Participants were categorized into three age groups (21-30, 31-40, and 41-50 years). Semen parameters, including sperm concentration, semen volume, motility, and morphology, were assessed according to the World Health Organization guidelines.

Results: Among the participants, no significant differences were observed in semen volume, motility, and morphology across different age groups. However, a statistically significant difference in sperm concentration among the three age groups was observed (p = 0.022). Spearman correlation analysis revealed a positive correlation between age and sperm concentration (r = 0.124, p = 0.013) as well as total sperm count (r = 0.10, p = 0.049).

Conclusion: In this study, no significant decline in semen quality with age was found among male partners of infertile couples aged 21-50 years. These findings highlight the complex relationship between age and semen quality and emphasize the need for further research to better understand the underlying mechanisms and provide more conclusive evidence regarding the impact of age on male fertility.

## Introduction

Infertility is a condition characterized by the inability of a sexually active couple to conceive after one year of regular, unprotected intercourse [1]. This definition recognizes that infertility affects both males and females and acknowledges that male factors contribute to approximately 40%-50% of all infertility cases, either alone or in combination with female causes [2,3]. While menopause signals the end of female reproductive capability typically between the ages of 45 and 55 years, males do not experience a definitive, unavoidable cessation of their reproductive potential [4].

However, it is crucial to investigate whether advanced paternal age is associated with diminished semen quality and an increased risk of infertility, as more males nowadays choose to father children at older ages [5]. Understanding the potential impact of advanced paternal age on male fertility and semen quality is essential, given this changing trend.

In the context of male infertility, defects in semen quality are often implicated, as the quality of semen serves as an important indicator of male fecundity [6]. Factors such as low sperm concentration, reduced sperm motility, and abnormal sperm morphology are primary contributors to male infertility [7]. Various factors, such as lifestyle choices, scrotal temperature, clothing, exposure to hot water, and the use of mobile phones, can have either positive or negative effects on the quality of semen [8,9]. However, previous studies have yielded inconsistent findings regarding the correlation between age and semen quality. Some studies indicate a negative correlation [10,11], while others suggest no significant association [12] or even a positive effect [13].

These discrepancies in findings could be attributed to various factors such as differences in study design, participant ethnicities, and geographical settings. Given the limited comprehensive data available on this subject in eastern India, our study aimed to address this knowledge gap by analyzing a cohort of male partners of infertile couples aged between 21 and 50 years.

## Materials and methods

In this cross-sectional observational study, we analyzed the records of 390 males who were referred to the andrology laboratory for semen quality assessment, as part of the routine evaluation of infertile couples, between January 2019 and December 2022. All those between the age of 21 and 50 years but with no known cause of infertility were included in the study. Individuals with urological disorders or any recognized factors causing infertility were excluded from the study. The study is part of a larger study, and prior informed consent was taken from all the participants. The study protocol and procedures were approved by the Institutional Ethics Committee of All India Institute of Medical Sciences (AIIMS) Patna (approval number: IEC2019372).

Semen analysis

On the first visit, after taking informed consent, the participants were advised to present to the laboratory after an abstinence period of 2-7 days. Semen samples from the participants were obtained through masturbation and collected in wide-mouthed sterile containers. The semen samples of participants who reported any spillage were discarded and advised to come again. The collected semen samples were weighed and kept at 37°C in an incubator for liquefaction. Semen analysis was done manually within one hour of collection as per the recommendations of the World Health Organization [14]. Two laboratory technicians were involved in the analysis of the semen samples using the same equipment. Regular internal quality control measures were implemented to avoid any significant discrepancies between results.

Statistical analysis

Statistical analyses were conducted using Statistical Package for the Social Sciences (SPSS) version 22.0 (IBM SPSS Statistics, Armonk, NY, USA). For the purpose of comparison, the participants were categorized into three age groups: 21-30 years, 31-40 years, and 41-50 years. The normality of the data within each group was assessed. Continuous parameters that followed a normal distribution are presented as means and standard deviations (SDs) and were compared using the one-way analysis of variance (ANOVA) test. Non-normally distributed continuous variables are presented as medians and interquartile ranges (IQRs) and were compared using the Kruskal-Wallis test. All categorical variables are presented as numbers and percentages. The Spearman correlation coefficient (r) was calculated to determine the linear association between age and the studied variables. A p-value of less than 0.05 was considered statistically significant.

## Results

Out of the total of 390 participants, 158 were in the age range of 21-30 years, 188 were in the age range of 31-40 years, and 44 were in the age range of 41-50 years. The demographic information of the participants is presented in Table 1, showing a statistically significant difference in body mass index (BMI) among the three age groups (p < 0.001). The mean BMI for each age group was as follows: 22.67 kg/m^2^ for the 21-30 years group, 24.57 kg/m^2^ for the 31-40 years group, and 25.08 kg/m^2^ for the 41-50 years group. The semen parameters of the different age groups are shown in Table 2, demonstrating a statistically significant difference in median sperm concentration among the three age groups (p = 0.022). The median sperm concentrations for each age group were as follows: 47.3 × 10^6^ cells/mL for the 21-30 years group, 70.3 × 10^6^ cells/mL for the 31-40 years group, and 81.3 × 10^6^ cells/mL for the 41-50 years group. However, no significant differences were observed in the abstinence period, semen volume, percentage motility, and sperm morphology among the different age groups The estimated Spearman correlation coefficients for various semen parameters with age are shown in Table 3, indicating statistically significant positive correlations between age and sperm concentration (r = 0.124, p = 0.013) and total sperm count (r = 0.1, p = 0.049).

**Table 1:** Distribution of participants in terms of demographics p-values were determined using the Kruskal-Wallis test or the ^a^one-way ANOVA test. ANOVA: analysis of variance, BMI: body mass index, SD: standard deviation, IQR: interquartile range

Parameter		Overall (N = 390)	Age group (years)			p-value
			21-30 (n = 158)	31-40 (n = 188)	41-50 (n = 44)	
Age (year) (median (IQR))		32.0 (9.0)	27.0 (3.0)	35.0 (5.0)	43.0 (5.0)	<0.001
BMI (kg/m^2^) (mean (SD))		23.86 (3.47)	22.67 (3.45)	24.57 (3.37)	25.08 (2.64)	<0.001^a^
Residence (frequency (%))	Rural	169 (43.3%)	81 (51.3%)	75 (39.9%)	13 (29.5%)	-
	Semi-urban	50 (12.8%)	20 (12.6%)	25 (13.3%)	5 (11.4%)	
	Urban	171 (43.8%)	57 (36.1%)	88 (46.8%)	26 (59.1%)	

**Table 2:** Comparison of semen parameters between different age groups Data are shown as median (IQR). p-values were determined using independent-samples Kruskal-Wallis tests. IQR: interquartile range

Parameter	Overall (N = 390)	Age group (years)			p-value
		21-30 (n = 158)	31-40 (n = 188)	41-50 (n = 44)	
Abstinence (day)	4.0 (2.0)	4.0 (2.0)	4.0 (2.0)	4.0 (3.0)	0.058
Semen volume (mL)	3.0 (2.0)	3.0 (2.0)	3.0 (1.5)	2.5 (1.5)	0.262
Sperm concentration (×10^6 ^cells/mL)	63.8 (70.8)	47.3 (64.3)	70.3 (69.9)	81.3 (101.9)	0.022
Total sperm count (×10^6^ cells)	162.5 (244.1)	133.1 (232.2)	175.8 (243.2)	205.4 (269.2)	0.065
Total motility (%)	66.0 (22.0)	66.5 (20.0)	67.0 (23.0)	59.0 (25.0)	0.708
Normal morphology (%)	7.0 (7.0)	8.0 (8.0)	7.0 (7.0)	7.0 (7.0)	0.062

**Table 3:** Correlation of different sperm parameters with age ^a^Analyzed by Spearman correlation analysis

Parameter	Correlation coefficient^a^	p-value^a^
Semen volume	-0.065	0.198
Sperm concentration	0.124	0.013
Total sperm count	0.10	0.049
Total motility	-0.033	0.516
Normal morphology	-0.018	0.729

## Discussion

It is generally observed that as males age, there is a tendency for a decline in semen quality. Our study aimed to investigate the relationship between age and semen parameters in male partners of infertile couples aged between 21 and 50 years. We analyzed samples from 390 such males who were referred to our laboratory between January 2019 and December 2022. Contrary to common observations, our findings did not indicate a decline in semen quality with age. In fact, we found no significant alterations in semen parameters associated with aging, except for an increase in sperm concentration.

Our results are in line with previous studies conducted by Andolz et al. [13] and Nieschlag et al. [15], which reported a positive correlation between increasing age and sperm concentration. Andolz et al. investigated a large sample of infertile males and reported a statistically significant increase of 0.7% in sperm concentration per year of age [13]. Similarly, Nieschlag et al. also observed a significant difference in sperm concentration between older and younger males, with older individuals displaying higher levels [15]. These studies, along with others, have documented linear increases in sperm concentration with age [16,17].

However, our findings diverge from studies that have reported a continuous decline in sperm concentration with age. Auger et al. documented a substantial decline in sperm concentration between the ages of 30 and 50 years [10]. Similarly, Haidl et al. found that the older age group (>45 years) had approximately half the sperm concentration compared to the younger group (<35 years) [18]. Furthermore, Kumar et al. conducted a study that demonstrated a decline in sperm concentration with increasing age in male partners of infertile couples at a rural tertiary care center in central India [11].

Several other studies have reported varied findings regarding the association between age and semen parameters in both healthy and infertile males. Brahem et al. observed that sperm concentration tends to increase with age among infertile males while remaining consistent in healthy males [19]. Plastira et al. also found significant increases in sperm concentration in oligo-astheno-teratozoospermic patients with age, while the control group of fertile males showed no significant changes [20].

The contradictory results regarding the impact of age on sperm concentration might be attributed to age-related alterations that can affect spermatogenesis. These alterations include diminished testicular perfusion, degeneration of germ cells, impaired function of Sertoli cells, and a reduction in Leydig cell quantity and functionality [21-23]. However, it is also plausible that an abnormal acceleration of spermatogenesis caused by impaired testicular receptivity to hormonal stimuli could lead to increased sperm concentration with age [24].

In our analysis, we did not find any statistically significant correlation between age and sperm motility, sperm morphology, or semen volume. These findings align with a study conducted by Berling et al., which also reported no significant association between these semen parameters and increasing male age [12]. Moreover, other researchers have documented similar results, indicating no significant association between age and sperm motility [25,26], normal morphology [26,27], and semen volume [25,28].

In contrast, Harris et al. reported a gradual decrease of 0.17% to 0.6% per year of age in terms of motility, 0.2% to 0.9% per year of age in normal sperm morphology, and 0.15% to 0.2% per year of age in semen volume [7]. Several other authors, including Kumar et al. [11] and Verón et al. [29], have reported a statistically significant association between declining sperm motility, sperm morphology, semen volume, and increasing male age. Additionally, Kidd et al. documented cumulative evidence from multiple studies supporting a decline in semen traits with advancing age, particularly among males aged 50 years and above [30].

Several studies examining various age groups have consistently reported diminished proportions of motile sperm in more advanced age categories [15,18,28]. Similarly, in studies categorizing age, a notable decrease of 4% to 22% has consistently been observed in the proportion of sperm with normal shapes among males aged 50 years and above compared to those aged 30 years and below [18,31]. Similar trends of decline beyond the age of 50 have also been documented in studies examining a decrease in semen volume [13,31].

The divergent findings between our study and other studies can be attributed to several factors, including disparities in study populations, sample sizes, geographical locations, and methodology. It is essential to consider these variations when interpreting and comparing the results of different studies. Additional research is warranted to further investigate the relationship between age and semen quality, accounting for these potential confounding factors.

## Conclusions

In conclusion, our study did not find evidence of deteriorating semen quality with age in male partners of infertile couples aged between 21 and 50 years. The relationship between age and semen parameters remains complex, with varying findings reported in the literature. Further research is required to elucidate the underlying mechanisms and provide more conclusive evidence regarding the impact of age on semen quality.
